# Role of mitochondria DNA A10398G polymorphism on development of Parkinson's disease: A PRISMA‐compliant meta‐analysis

**DOI:** 10.1002/jcla.24274

**Published:** 2022-02-11

**Authors:** I‐Shiang Tzeng

**Affiliations:** ^1^ Department of Research Taipei Tzu Chi Hospital Buddhist Tzu Chi Medical Foundation New Taipei City Taiwan

**Keywords:** allele, mitochondrial DNA, neurogenesis, Parkinson's disease

## Abstract

**Background:**

Parkinson's disease (PD) is characterized by memory loss and multiple cognitive disorders caused primarily by neurodegeneration. However, the preventative effects of the mitochondrial A10398G DNA polymorphism remain controversial. This meta‐analysis comprehensively assessed evidence on the influence of the mitochondrial DNA A10398G variant on PD development.

**Methods:**

The PubMed, EMBASE, EBSCO, Springer Link, and Web of Science databases were searched from inception to May 31, 2020. We used a pooled model with random effects to explore the effect of A10398G on the development of PD. Stata MP version 14.0 was used to calculate the odds ratios and 95% confidence intervals (CIs) from the eligible studies to assess the impact of mitochondrial DNA A10398G on PD development.

**Results:**

The overall survey of the populations showed no significant association between mitochondrial DNA A10398G polymorphism (G allele compared to A allele) and PD (odds ratio = 0.85, 95% CI = 0.70–1.04, *p* = 0.111); however, a significant association between the mutation and PD was observed in the Caucasian population (odds ratio = 0.71, 95% CI = 0.58–0.87, *p* = 0.001). A neutral effect was observed in the Asian population (odds ratio = 1.10, 95% CI = 0.94–1.28, *p* = 0.242).

**Conclusions:**

The results of this meta‐analysis showed the potential protective effect of the mitochondrial DNA A10398G polymorphism on the risk of developing PD in the Caucasian population. Studies with better designs and larger samples with intensive work are required to validate these results.

## Graphical abstract

Parkinson's disease (PD) is characterized by memory loss and multiple cognitive disorders caused primarily by neurodegeneration. Neurodegeneration is the major cause of symptoms. A previous genome‐wide association study of PD identified many genetic variants in genes including PARKIN, GBA, LRRK2, and SNCA associated with PD. Moreover, mutations in mitochondria‐related genes, including PARKIN, PARK6, POLG, DJ, and 12S rRNA are also implicated in PD. While most of these genes are nuclear, the 12S rRNA gene is mitochondrial.

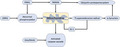

## 1 INTRODUCTION

Parkinson's disease (PD) is characterized by memory loss and multiple cognitive disorders.^1^ Neurodegeneration is the major cause of symptoms.^2^ A previous genome‐wide association study of PD^3^ identified many genetic variants in genes including *PARKIN*, *GBA*, *LRRK2*, and *SNCA* associated with PD. Moreover, mutations in mitochondria‐related genes, including *PARKIN*, *PARK6*, *POLG*, *DJ*, and 12S rRNA are also implicated in PD.^4^, ^5^ While most of these genes are nuclear, the 12S rRNA gene is mitochondrial. To build (or maintain the function of) mitochondria,^6^ these relevant genes are involved in encoding mitochondrial proteins.

Mitochondria play a critical role in providing cellular energy, metabolism regulation, cell death, etc. Various diseases may be caused by mitochondrial dysfunction. Owing to mitochondrial dysfunction associated with PD, sporadic PD patients may have respiratory chain enzyme deficiencies in the substantia nigra (SN).^7^, ^8^, ^9^ Thus, the elucidation of the mechanism of genetic influence requires exploration of the genetic components of PD. PD is a common phenotype of several diseases, a critical finding in the past decade. As PD is genetically heterogeneous, various genes can contribute to its development. While most causes of PD development are multifactorial, monogenic forms related to mutations in genes like *PARKIN* and *LRRK2* account for 10% of PD cases. Thus, different genes are important for PD development in most cases. Some genes are more likely than others to influence the risk of an individual developing PD. This genetic heterogeneity may provide conflicting information and confusing data in research studies.

Sharing a point mutation with a single‐nucleotide polymorphism (SNP),^10^ a group of similar haplotypes forms a haplogroup. Alleles located in different chromosomal regions that are combined as a haplogroup are closely connected and inherited together. Different types of mainstream Y‐DNA haplogroups are detected in different races. For example, the H, I, J, K, T, U, V, W, and X haplogroups are detected in European races.^11^ Different haplogroups may confer increased (or decreased) risks of PD development. van der Walt et al.^12^ reported a reduced PD risk for the J and K haplogroups, especially in women (609 affected, 340 controls). Subsequent studies with Irish and Finnish populations^13^, ^14^ with small sample sizes (90 cases vs. 129 controls and 238 cases vs. 104 controls, respectively) showed that the J and T haplogroups were associated with increased PD risks. Recently, a study with a larger sample size (455 affected vs. 447 nonaffected participants) by Pyle et al.^15^ showed that the U, K, J, and T haplogroups were related to a reduced risk for PD development, consistent with the findings reported by van der Walt.

van der Walt et al.^12^ and Pyle et al.^15^ reported a significantly reduced risk (*p* < 0.0001) for the U, K, J, and T haplogroups (1302 affected, 891 controls). van der Walt attributed this observation to the A10398G SNP polymorphism. Pyle et al. suggested that other SNPs might be causative, as this SNP was also found in other haplogroup backgrounds.^15^ Furthermore, another small study on the association of A10398G with PD (102 affected, 112 controls) observed no significant relationship with PD.^16^

While several recent studies concluded that the mitochondrial A10398G DNA polymorphism prevented PD,^12^, ^17^, ^18^ other studies did not.^16^, ^19^, ^20^, ^21^, ^22^, ^23^, ^24^ Thus, the present meta‐analysis evaluated 10 eligible case‐control studies to provide comprehensive evidence of the influence of the mitochondrial A10398G DNA variant on the development of PD.

## 2 MATERIALS AND METHODS

### 2.1 Relevant and eligible literature search

Two investigators independently searched the PubMed, EMBASE, EBSCO, Springer Link, and Web of Science databases for relevant literature published through May 2020 using the following keywords: mitochondrial DNA or A10398G, gene polymorphism or variant, and familial Parkinson's disease or familial PD. Human case‐control studies set to be searched as references.

### 2.2 Inclusion and exclusion criteria for the eligible studies

The case‐control studies included in this analysis fulfilled four inclusion criteria: (1) explored the effect of mitochondrial DNA polymorphism A10398G on PD, (2) provided odds ratios (ORs) and 95% confidence intervals (CIs) from sufficient allele data, (3) investigated equal numbers of cases and controls, and (4) were written in English and had available full texts.

The exclusion criteria were: (1) case reports, editorials, review articles, or meta‐analyses; and (2) investigations of mitochondrial DNA polymorphism A10398G without sufficient data.

### 2.3 Extraction and assessment of the eligible studies

Two investigators (IST and an anonymous investigator) independently assessed eligible articles and collected the required data. We also discussed with a third reviewer (second anonymous investigator) to resolve disagreement between the two investigators. We extracted and presented the eligible studies as the surname of the first author followed by et al., and recorded the publication year, population ethnicity, and the number of cases and controls. The quality of the eligible studies was evaluated independently by IST and the anonymous investigators according to the Newcastle–Ottawa Scale (NOS).^25^ We identified articles of high quality, defined as NOS scores ≥6 stars.

### 2.4 Ethical statements

This was a literature‐based study; thus, no ethical approval was required.

### 2.5 Statistical analysis

Stata MP, version 14.0 (Stata Corporation) was used to analyze the extracted data. To assess the impact of the relationship between the variant and PD sensitivity, OR and CI were calculated. Statistically significant differences were defined as *p* < 0.05. Q‐ and I^2^ tests were used to examine the heterogeneity between studies.^26^ Furthermore, the relationship between the mitochondrial A10398G DNA variant and PD risk was assessed using the random‐effects model of allele data.^27^ We also addressed concerns regarding heterogeneity through subgroup analysis stratified by different populations to re‐examine the relationship between mitochondrial A10398G DNA variants and PD risk. In addition, we conducted a sensitivity analysis to examine the stability of the results. Funnel plots and Begg's tests were used to explore potential publication bias. We concluded that no trial publication bias was embedded in the meta‐analysis (*p *> 0.05).^28^

## 3 RESULTS

**FIGURE 1:**
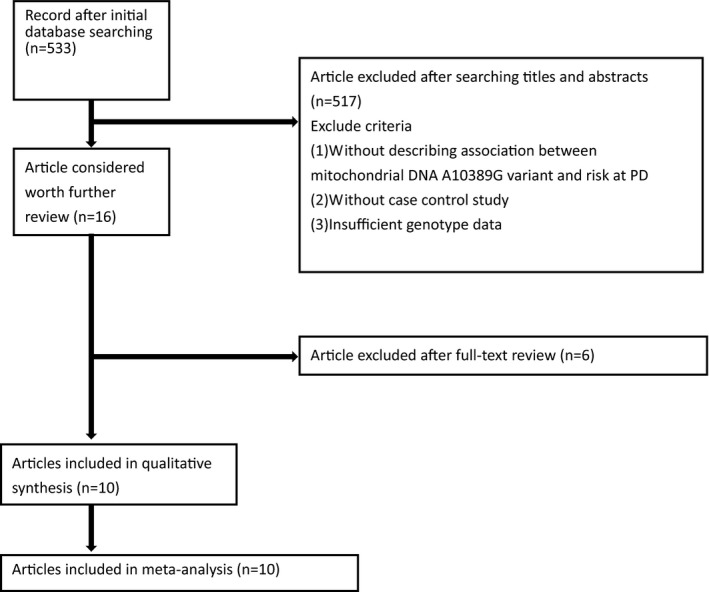
Flow chart for publication selection

Figure 1 illustrates the article screening process. First, the database search identified 533 potentially relevant articles. The mitochondrial DNA variant in familial PD was also crucial for the OR of the G allele of the pooling model. This study analyzed a total of 3809 PD patients and 3240 healthy individuals. Among the included studies, seven and three assessed Caucasian and Asian populations, respectively. Among the eligible studies, eight and two used polymerase chain reaction (PCR) or TaqMan, respectively, to measure the genotype frequencies of the controls. Tables S1 and S2 show the relevant characteristics and NOS scores for each eligible study, respectively.

### 3.1 Main findings

**FIGURE 2:**
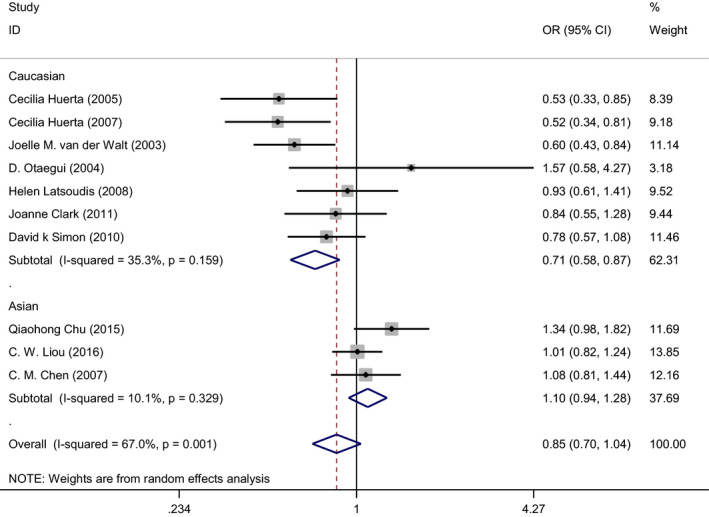
Forest plot for the present meta‐analysis

Table 1 summarizes the results of the meta‐analysis. The I^2^ value indicated the presence of heterogeneity in the allele model under the random‐effects assumption (I² = 67.0%, *p* = 0.001). The results of a subgroup analysis stratified by Caucasian (I² = 35.3%, *p* = 0.159) and Asian (I² = 10.1%, *p* = 0.329) populations showed no significant relationship between the mitochondrial A10398G DNA polymorphism and PD (G allele compared to A allele: OR = 0.85, 95% CI = 0.70–1.04, *p *= 0.111) in the pooled populations. However, individually, we observed a significant relationship between this polymorphism and PD in the Caucasian population (G allele compared to A allele: OR = 0.71, 95% CI = 0.58–0.87, *p* = 0.001) but not in the Asian population (G allele compared to A allele: OR = 1.10, 95% CI = 0.94–1.28, *p *= 0.242). Figure 2 presents forest plots of the included studies.

**TABLE 1:** Summary of the results of the meta‐analysis

Ethnicity	Fix effect pooling model (Mantel‐Haenszel)			Random effect pooling model (Mantel‐Haenszel heterogeneity)			Heterogeneity	Begg's test	
	OR	95% CI	*p*	OR	95% CI	*p*	I^2^	Z	*p*
Overall population	0.89	0.80–1.00	0.041	0.85	0.70–1.04	0.111	67.0%	0.72	0.474
Caucasian population	0.71	0.61–0.83	<0.001	0.71	0.58–0.87	0.001	35.3%	0.00	1.000
Asian population	1.09	0.94–1.27	0.231	1.10	0.94–1.28	0.242	10.1%	1.04	0.296

Abbreviations: CI, confidence intervals; OR, odds ratio.

### 3.2 Sensitivity test and publication bias of the included studies

**FIGURE 3:**
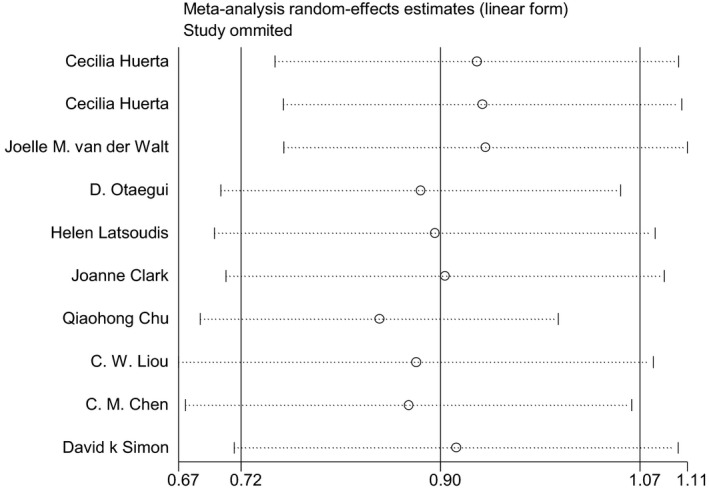
Sensitivity analysis of the summary odds ratio coefficients on the association between mitochondrial DNA A10398G polymorphism and PD

**FIGURE 4:**
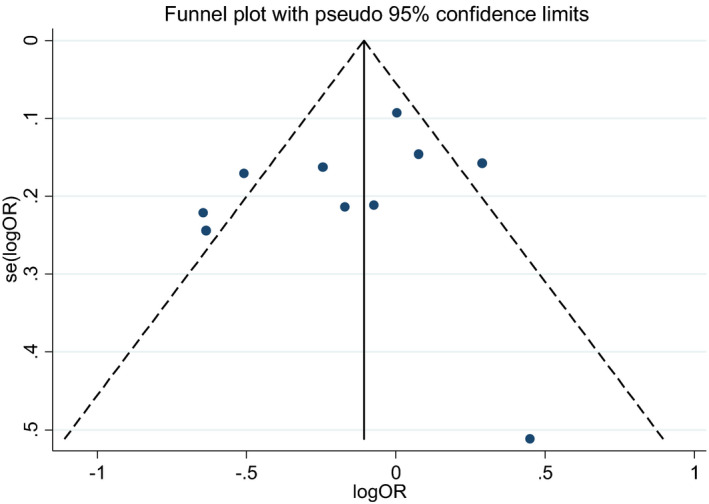
Funnel plot

In the allele model, the sensitivity tests for integrated OR and CI showed no significant changes when removing each study individually. The OR of the meta‐analysis also remained stable (Figure 3). Figure 4 shows Begg's funnel plots indicating no publication bias in this meta‐analysis (*p* = 0.474).

## 4 DISCUSSION

Endurance training is a powerful tool to improve health and performance. Physical activity is an effective intervention for many diseases.^29^, ^30^ Activating mitochondrial function and exercising muscles are among the most profound adaptations.^31^ Physical activity is positively associated with mitochondrial quality and quantity, and positive health effects have been reported after endurance training. Furthermore, muscle oxidative capacity and endurance performance are strongly associated with high mitochondrial content.

PD is one of the most common neurodegenerative disorders due to the characteristic loss of dopaminergic neurons in the SN. Although the genetic factors involved in the development of PD have been identified, some etiological factors of PD remain ambiguous. Previous studies showed that mitochondrial dysfunction triggers PD development.^32^, ^33^, ^34^ Other neurodegenerative disorders, including Alzheimer's disease, Lewy body disease, and amyotrophic lateral sclerosis, as well as multiple sclerosis, also involve mitochondrial DNA mutations.^35^, ^36^, ^37^ Based on our findings, the effect of the mitochondrial A10398G DNA polymorphism on PD development remains to be elucidated. Although previous studies evaluated the effects of polymorphisms on PD sensitivity in various populations worldwide, their results were contradictory. The results of this study successfully clarified the effect of mitochondrial DNA A10398G on PD development and addressed the limitations of the relatively small sample sizes in some of the included studies.

Among included case‐control studies, seven and three investigated Caucasian and Asian populations, respectively. Among 271 cases and 230 controls in the Spanish population, Huerta et al.^17^ reported a decreased risk of PD for the 10398G allele, with an OR (95% CI) of 0.53 (0.33–0.86).^17^ Another study including 450 cases and 200 controls in a Caucasian population from Spain^18^ reported a decreased risk of PD for the 10398G allele, with an OR (95% CI) of 0.52 (0.34–0.81).^18^ Similarly, van der Walt demonstrated the strong protective effect of 10398G against PD in European populations, with an OR (95% CI) of 0.53 (0.39–0.73).^12^ However, Chu et al. reported negative associations, with 10398G allele a significant and positive risk factor for PD in a northern Chinese population (OR = 1.30; 95% CI = 0.95–1.76; *p* = 0.013).^24^ Other studies observed no statistically significant link between the 10398G allele and PD risk. For instance, Otaegui et al. found no relationship between the A10398G polymorphism and PD in the general Caucasian population of Spain.^16^ Clark et al. also detected no relationship between A10398G and PD risk in a Caucasian population.^19^ Similarly, a case‐control study including 416 cases and 372 controls by Chen et al. detected no association between A10398G and PD.^20^ Moreover, a case‐control study by Latsoudis et al.^21^ of 224 cases and 383 controls and a study by Liou et al.^22^ including 725 cases and 744 controls reported similar G allele frequencies among cases and controls. The random‐effects results of the present meta‐analysis showed no increased risk of PD for the G allele compared to the A allele (OR = 1.1, 95% CI = 0.94–1.28, *p* = 0.242) in the Asian population, consistent with the findings of the included studies. Meanwhile, compared to the A allele, the G allele of the mitochondrial A10398G DNA polymorphism exhibited a protective function against PD (OR = 0.71, 95% CI = 0.58–0.87, *p* = 0.001) in the Caucasian population.

Due to the amino acid replacement from Thr to Ala, which leads to a transition from A to G, the A10398G ND3 SNP may encode one subunit constituting complex I.^38^ The mitochondrial electron transport is catalyzed by a very large enzyme complex I.^39^, ^40^ The trivially correlated 10398G allele may improve the expression of complex I.^12^ An active complex I may lead to increased ATP synthesis and defend against various PD‐related biotoxins.^41^ Furthermore, the 10398G allele may result in increased reactive oxygen species (ROS) compared to the 10398A allele. ROS are produced by complex I during cellular activity.^42^ However, alleviating oxidative stress enhances complex I and decreases ROS generation.^43^ In general, SN tissues are vulnerable to oxidative damage; thus, SN neuron degeneration may be caused by excessive oxidative stress over time.^44^ Therefore, the 10398G allele may have neuroprotective functions to reduce the risk of PD. More evidence‐based research is required to elucidate the mechanism underlying the regulation of complex I.

The results in the Caucasian population differed from those of the Asian population due to the different distributions of the A10398G polymorphism. A previous study showed a lower frequency of the 10398G mitochondrial DNA allele in two Asian populations compared to that of a Caucasian population.^45^ Our meta‐analysis results were similar to those of a previous meta‐analysis.^46^ Unlike the meta‐analysis by Hua et al.,^46^ our study additionally included the study by Simon et al.^23^ because PD includes familial and sporadic forms. Caucasian and Asian populations may also exhibit contradictory findings due to their different exposures to environmental factors. The etiology of PD is still largely unknown; however, the condition is likely to be multifactorial, with genetic and environmental factors contributing to disease genesis. Numerous environmental toxins have been implicated in the onset of PD. Moreover, the incidence of PD may be associated with occupational exposure to chemicals.^47^ Individuals exposed to pesticides^48^ and heavy metals^49^ may also have increased risks of PD. Highly populated urban areas also showed significant associations of airborne heavy metal pollution from industry^50^ and traffic ambient air pollution^51^ with increased risk of PD onset. A previous study suggested that mainland Chinese has a lower rate of exposure to putative environmental risk factors for PD compared to the West.^52^ Hence, larger samples and well‐designed studies are required to validate these results.

Randomized clinical trials provide research inference with the highest level of evidence. However, some factors, including environmental and socioeconomic factors, may not be completely excluded to strictly control for their impact in epidemiologic research. Regardless of how perfect the design and measurement of epidemiologic research are, it is impossible to exclude potential and unmeasured confounders. Hence, although the research designs may be similar, they produce different conclusions, eventually leading to controversy. Meta‐analyses are commonly used to address controversial conclusions from relevant studies on similar issues. After considering the weight (random‐effects model), the pooled model provides greater objective research results. Recently, Mendelian randomization was used to modify potential and unmeasured confounders to obtain true causal inferences. Billingsley et al. used this method to identify 14 mitochondrial function‐associated genes linked with the risk of PD.^53^ In future, superior to both research approaches, a Mendelian randomization meta‐analysis may help to identify the relationships between specific mitochondrial DNA polymorphisms and PD.

This meta‐analysis has some limitations. First, PD development may also be influenced by the interactions of genetic‐genetic or genetic‐environmental factors. Second, the small sample sizes may have limited the reliability of the studies. In particular, only three studies included an Asian population. Furthermore, due to insufficient data, other possible variables associated with A10398G and PD, including age of onset, sex, and smoking, were not assessed.

## 5 CONCLUSION

The mitochondrial A10398G DNA polymorphism plays a protective role in the risk of PD development in the Caucasian population. These results require validation in well‐constructed studies with larger sample sizes.

## CONFLICT OF INTEREST

The author declared no competing interests.

## AUTHOR CONTRIBUTIONS

The author constructed all works of the final manuscript. I‐Shiang Tzeng involved in the conceptualization, formal analysis, methodology, supervision, writing original draft, reviewing and editing. I‐Shiang Tzeng and anonymous investigators involved in the data curation.

## Supporting information

Table S1‐S2

## Data Availability

The data that support the findings of this study are available from the corresponding author upon a reasonable request.
