# Supplementary material for: Role of mitochondria DNA A10398G polymorphism on development of Parkinson's disease: A PRISMA‐compliant meta‐analysis
Source: J Clin Lab Anal. 2022 Feb 11;36(3):e24274. doi: 10.1002/jcla.24274 (PMC8906025; doi:10.1002/jcla.24274)
Supplement: Supplementary file 1 — Table S1‐S2 [file JCLA-36-e24274-s001.docx]

Table S1. The relevant characteristics of each eligible study

| First author followed by et al. | Year | Ethnicity | Genotyping method | Sample size | Allele distribution |  |  | Relation |
| --- | --- | --- | --- | --- | --- | --- | --- | --- |
|  |  |  |  |  | G | A | Chi-square |  |
| Huerta et al. | 2005 | Caucasian | PCR-RFLP | 271/230 | 34/49 | 237/181 | 0.0121 | Increased |
| Huerta et al. | 2007 | Caucasian | PCR-sequencing | 450/200 | 58/44 | 392/156 | 0.0046 | Increased |
| Chen et al. | 2007 | Asian | PCR-RFLP | 416/372 | 257/223 | 159/149 | 0.6504 | Insignificant |
| Liou et al. | 2016 | Asian | SPCR | 725/744 | 725/744 | 328/338 | 0.131 | Insignificant |
| Simon et al. | 2010 | Caucasian | Taqman | 428/430 | 89/108 | 339/322 | 0.1545 | Insignificant |
| Otaegui et al. | 2004 | Caucasian | PCR-sequencing | 40/64 | 9/10 | 31/54 | 0.534 | Insignificant |
| Latsoudis et al. | 2008 | Caucasian | PCR-RFLP | 224/383 | 43/78 | 181/305 | 0.8083 | Insignificant |
| Clark et al. | 2011 | Caucasian | PCR-RFLP | 376/173 | 84/44 | 292/129 | 0.4917 | Insignificant |
| van der Walt et al. | 2003 | Caucasian | Taqman | 557/312 | 97/81 | 460/231 | 0.0036 | Increased |
| Chu et al. | 2015 | Asian | PCR-RFLP | 322/332 | 186/168 | 136/164 | 0.07857 | Insignificant |

Table S2. The Newcastle-Ottawa Scale assessed for each eligible study

| Ref. no | First author followed by et al (Year) | Selection (stars) | Comparability (stars) | Exposure (stars) | Total Quality score |
| --- | --- | --- | --- | --- | --- |
| [16] | Huerta et al. (2005) | 4 | 2 | 1 | 7 |
| [17] | Huerta et al. (2007) | 4 | 2 | 1 | 7 |
| [19] | Chen et al. (2007) | 4 | 2 | 1 | 7 |
| [21] | Liou et al. (2016) | 3 | 2 | 1 | 6 |
| [22] | Simon et al. (2010) | 4 | 2 | 1 | 7 |
| [15] | Otaegui et al. (2004) | 4 | 2 | 1 | 7 |
| [20] | Latsoudis et al. (2008) | 4 | 2 | 1 | 7 |
| [18] | Clark et al. (2011) | 3 | 2 | 1 | 6 |
| [11] | van der Walt et al. (2003) | 3 | 2 | 1 | 6 |
| [23] | Chu et al. (2015) | 4 | 2 | 1 | 7 |
